# Renal and cardiovascular prognostic significance of echocardiographic early diastolic mitral annular velocity in IgA nephropathy

**DOI:** 10.1007/s10554-023-02988-7

**Published:** 2023-11-07

**Authors:** Balázs Sági, István Késői, Tibor Vas, Botond Csiky, Judit Nagy, Tibor Kovács

**Affiliations:** 1 2nd Department of Internal Medicine and Nephrology, Diabetes Center, Medical School, Clinical Center, University of Pécs, Pacsirta Street 1., Pécs, 7624 Hungary; 2 Fresenius Medical Care Dialysis Center Pécs, Pécs, Hungary; 3 Department of Internal Medicine and Cardiology, Hospital of Mohács, Mohacs, Hungary

**Keywords:** Cardiovascular risk, Chronic kidney disease, Diastolic dysfunction, IgA nephropathy, Tissue Dopper imaging echocardiography

## Abstract

In chronic kidney disease (CKD), as in IgA nephropathy (IgAN), cardiovascular (CV) mortality and morbidity are many times higher than in the general population, and diastolic dysfunction (LVDD) has prognostic significance as well. Tissue Doppler Echocardiography (TDI) is another method for measuring myocardial contractility and determining diastolic dysfunction. 79 IgAN patients (age 46 ± 11 years) with CKD stages 1–3 were investigated and followed for 70 ± 28.7 months. Doppler echocardiography was used to measure the E (early) and A (late) waves, as well as the E wave deceleration time (EDT) during mitral inflow. TDI was used to measure early (Ea) and late (Aa) diastolic velocities (lateral and septal basal wall fragment average). From these, we calculated the E/Ea and Ea/Aa ratios. The primary combined endpoints were total mortality, major CV events, and end-stage renal disease, and the secondary endpoints were cardiovascular or renal (eGFR decreased below 15 ml/min/1.73 m^2^ or renal replacement therapy was started). Patients with decreased Ea (< 13 cm/s) had significantly more endpoints (20/42 vs. 3/37; p = 0.001) than patients with higher Ea (≥ 13 cm/s). The secondary renal endpoints were also significantly higher (p = 0.004). In a multivariate model, the eGFR showed independent correlation with the E/A ratio (r = 0.466; p < 0.01), EDT (r = − 0.270; p < 0.01), Ea/Aa ratio (r = 0.455; p < 0.01), and decreased Ea (r = 0.544; p < 0.01). Independent factors influencing Ea were only EDT by uni- and multivariate regression but age and albuminuria by logistic regression. Decreased Ea measured by TDI seems to be an eligible factor to predict the prognosis of IgA nephropathy. The decreased Ea may be a helpful parameter to identify high-risk CKD patients.

## Introduction

Based on former large-scale studies, cardiovascular (CV) mortality and morbidity are many times higher in chronic kidney disease (CKD) than in the general population, causing a public health issue worldwide [1, 2]. For patients of young ages (25- to 34-year-old) with end-stage kidney disease, annual mortality is increased 500- to 1000-fold and corresponds to that of the patients above the 80-year-old general population [3, 4]. In the case of GFR decline, the total mortality hazard ratio is 5.9, and the CV events ratio is 3.4 higher in ESKD than in GFR > 60 ml/min/1.73 m^2^ [5, 6]. All of these increased CV risks are caused by more common traditional and nontraditional risk factors in patients with CKD [7]. The remodeling of the myocardium and blood vessels could be one of these risk factors leading to CV events, heart failure, and progression to end-stage kidney disease (ESKD). Therefore, identifying these risk factors and high-risk patients is very important for interventional strategies and managing patients with CKD.

Echocardiography is a widely used and valuable noninvasive method for the determination of the left ventricular systolic, measuring systolic ejection fraction (LVEF), and diastolic function (LVDD), which has prognostic significance in ischemic heart disease, heart failure, and end-stage renal failure [8–11]. Tissue Doppler Imaging (TDI) echocardiography is another way to measure the rate of myocardial contractility and help refine diastolic dysfunction. In community-based epidemiological studies, the ratio of E (transmitral E wave velocity) to Ea (early diastolic mitral velocity) has been reported to be significantly associated with LV diastolic function and filling pressure [12].

CKD patients have cardiovascular disease more than twice as often as non-CKD patients. According to the USRDS Annual Data Report, nearly 40 percent of patients with stage 4–5 CKD carried a diagnosis of HF in 2015, according to the latest study showing 41–50 percent [13, 14]. Data regarding the prevalence of heart failure with preserved ejection fraction (HFpEF) in dialysis are scarce and are usually underdiagnosed. Contrary to heart failure with reduced ejection fraction (HFrEF), HFpEF is more prevalent in women and older patients. Over-65 patients account for more than half of all HF cases in the community [15]. In the highest age decile (≥ 90 years old), nearly all patients with HF have preserved EF. Studies focusing on defining the percentage of patients with HFpEF vs. HFrEF incidents or prevalent HF cohorts have demonstrated that approximately half of the patients with HF have HFpEF [16]. Due to a variety of factors, CKD and HFpEF are becoming more prevalent. Whether due to a common etiology or arising independently, CKD and HFpEF are often coincident in patients. Furthermore, the population with both problems is expanding. A study published a decade ago found that renal dysfunction is associated with worse outcomes and higher mortality in HFpEF patients. Despite the association between CKD and adverse outcomes, the interaction between CKD, clinical features, and cardiac structural and functional abnormalities in HFpEF has not been fully understood [17].

## Objective

The literature contains limited data about the prognostic effect of TDI parameters on cardiovascular and renal outcomes in CKD. The study's goal was to determine the predictive value of TDI parameters in a homogeneous group of IgAN patients with CKD stages G–3.

## Materials and methods

### Patients

We monitored 79 patients with IgAN at the University of Pécs' Clinical Center's 2nd Department of Internal Medicine, Nephrology, and Diabetes for an average of 70 months between 2009 and 2018. The diagnosis of IgAN was confirmed by renal biopsy in all patients. The local ethics committee approved the study protocol, and all participants gave their written consent to its completion. (Approval number: 3170/2008).

At the start of patient enrollment, echocardiography was performed, and classic CV risk factors (hypertension, carbohydrate metabolism disorder, obesity, lipid abnormalities, smoking) and patient medication were also recorded. The criteria of the ATP III (Adult Treatment Panel III) were used to identify the metabolic syndrome. The obesity criteria were a BMI over 30 kg/m^2^. Renal function was calculated using the CKD-EPI formula (eGFR, ml/min, 1.73 m^2^). Patients with severe comorbidities (active cancer treatment, fever, and kidney transplant recipients) were excluded. Renal replacement therapy, end-stage renal disease (CKD-5), and a history of kidney transplantation were also exclusion criteria. A 24-h blood pressure monitor was used by Meditech ABPM devices to determine the patient’s 24-h average systolic and diastolic blood pressure, pulse pressure, and diurnal index. Additional CV examinations (ergometry, coronagraphy, etc.) were also performed based on the patient's complaints.

Patients were observed regularly, and follow-up exams were performed every 3–6 months (more frequently if necessary). During these visits, medical events that had occurred since the previous visit were discussed, as well as physical status and detailed laboratory tests. Blood pressure values were determined from the average of three measurements taken after 10 min of rest.

The study's primary composite endpoints included cardiovascular outcomes, overall mortality, coronary intervention (due to an acute coronary event or acute myocardial infarction), stroke, and renal outcomes, such as the development of ESKD (eGFR decreased below 15 ml/min/1.73 m^2^ or renal replacement therapy was started). Then, the CV (overall mortality, coronary intervention, stroke) and renal endpoints (eGFR decreased below 15 ml/min/1.73 m^2^ or renal replacement therapy was started) were individually examined as secondary endpoints.

### Echocardiographic measurement

**Fig. 1:**
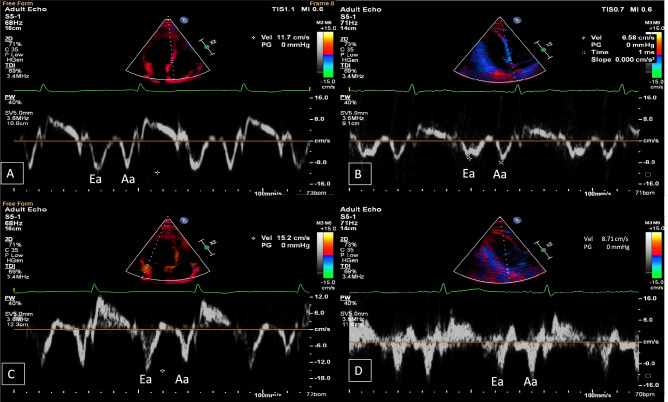
High and low early (Ea) and late diastolic mitral annular velocity (Aa) at the interventricular septal annulus (**A**, **B**) and lateral side of the mitral annulus (**C**, **D**) measured by tissue Doppler echocardiography

The Aloka SSD 1400 echocardiography equipment was used. Using 2D images of the length of the apical left ventricular segment and the area of the left ventricular short-axis muscle, the left ventricular mass (LVM) was computed (LVM = (5/6 area * length)). The Cornell criterion was used to determine LVMI, which was then indexed for height (in meters). The left ventricular ejection fraction (LVEF) was calculated by adding the diastolic and systolic volumes of the left ventricle using the unidirectional (Simpson method: EF = (Dvol-Svol) / Dvol * 100)). Based on traditional spectral Doppler measurements, mitral inflow and pulmonary venous flow were used to evaluate diastolic function. We also calculated the isovolumetric relaxation time (IVRT), the E wave deceleration time, and the E wave to A wave ratio (E/A ratio). RWT and/or LVMI abnormalities were used to define LVH. TDI was used to measure the early and late displacements of the lateral and septal basal wall fragments closest to the left ventricle (Ea and Aa) and calculate the average (Fig. 1). Then determine the E/Ea and Ea/Aa ratios. To exclude interindividual differences, two investigators (cardiologist specialists) examined all patients.

### Statistical analysis

We divided our patients into two groups according to the Ea cutoff (13 cm/s) for statistical analysis. Unless otherwise specified, all values are the mean and standard deviation. Differences between the two groups were compared by the Student’s t test, the Mann–Whitney U test for continuous variables, and the χ^2^ test for categorical variables. A bivariate correlation method (Pearson’s correlation) assessed the relationship between two continuous variables. The factors influencing Ea were investigated using univariate and multivariate linear regression analysis. We also performed univariate and multivariate logistic regression analyses to examine the relationships between Ea and other covariates. Survival was assessed by the Mantel-Cox log-rank test. The effect of factors influencing survival was analyzed by Cox regression analysis**.** SPSS version 22.0 (Statistical Program for Social Sciences for Windows, SPSS Inc., Chicago, IL) was used to analyze the data, and a significance level of 0.05 was used for statistical analysis. Values of p < 0.05 were considered statistically significant.

## Results

Based on our results, to determine the best cutoff values of Ea, we made a logistic regression analysis (ROC curve). 13 cm/s was the best cutoff to predict primary and secondary endpoints. The patients were divided into two groups based on this cutoff. The baseline characteristics showed significant differences in age, blood pressure, metabolic parameters (hypertension, dyslipidemia, BMI, carbohydrate metabolism disorder), eGFR, antihypertensive drugs (ACE/ARB, BB, CCB), and statin usage. The two groups had no significant difference in LV ejection fraction, LV end-diastolic diameter, hemoglobin, or lipid levels. However, the lower Ea group had significantly higher diastolic dysfunction, proteinuria, and uric acid levels (Table 1).

**Table 1:** Baseline characteristics of IgAN patients

Clinical data (n = 79)	Ea ≥ 13 (cm/s) (n = 37)	Ea < 13 (cm/s) (n = 42)	P
Man/woman (n/%)	21/16 (57/43)	29/13 (69/31)	NS
Age (year)	39.8 ± 11.2	52.1 ± 7.7	< 0.001
Average systolic/diastolic RR (Hgmm)	117/70 ± 11/68	127/76 ± 16/9	0.001
24h pulse pressure (Hgmm)	47.3 ± 12.8	50.7 ± 7.7	NS
Diurnal index systolic (%)	10.9 ± 6.2	10.3 ± 5.2	NS
Metabolic parameters			
Hypertension (n, %)	20 (54)	40 (95)	< 0.001
BMI (kg/m^2^)	26 ± 4.7	27.9 ± 4.5	0.015
Dyslipidaemia (n, %)	10 (27)	26 (62)	< 0.001
DM (n, %)	6 (16)	19 (45)	0.002
eGFR (ml/min)	103.7 ± 27.8	72.7 ± 29.6	< 0.001
Duration of kidney disease (year)	10 ± 9	11.5 ± 10	NS
Smoking (n, %)	6 (16)	8 (19)	NS
Metabolic syndrome (n, %)	5 (15)	17 (40)	0.003
Therapy			
ACEI/ARB (n, %)	24 (65)	41 (98)	< 0.001
BB (n, %)	6 (16)	16 (38)	0.015
Statin (n, %)	7 (19)	19 (45)	0.049
CCB (n, %)	7 (19)	15 (36)	0.049
Ergometry			
Average heart rate (beat/min)	73 ± 8.4	74 ± 8.8	NS
Stress test time (s)	618 ± 174	479 ± 178	< 0.001
HRR (bpm)	28.2 ± 8.7	21.3 ± 11.2	0.001
CAD (Positive stress test)	3 (8)	7 (16)	NS
Echocardiographic parameters			
LVEF (%)	62.8 ± 4.9	62.1 ± 7.7	NS
LVMI	94.5 ± 16	117.3 ± 23	< 0.001
LVM (g)	180.1 ± 44.0	225.8 ± 48.8	< 0.001
LVEDD (cm)	4.88 ± 0.39	5.02 ± 0.41	NS
DD (n/%)	8 (22)	29 (69)	< 0.001
E/A	1.26 ± 0.32	0.87 ± 0.24	< 0.001
EDT (ms)	178.7 ± 33.8	204 ± 43.5	0.003
Ea (cm/s)	16.6 ± 2.13	10.4 ± 2.08	< 0.001
Aa (cm/s)	10.75 ± 3.41	11.8 ± 2.63	NS
Ea/Aa	1.67 ± 0.54	0.93 ± 0.32	< 0.001
E/Ea	4.31 ± 0.95	5.38 ± 1.40	0.002
Laboratory results			
Hb (g/dl)	13.9 ± 1.56	13.4 ± 1.54	NS
Urine albumin (mg/day)	317.1 ± 550.9	632.2 ± 721.8	0.016
UA (umol/l)	288.4 ± 76.7	360.5 ± 68.8	0.015
Total cholesterol (mmol/l)	4.92 ± 0.95	5.12 ± 1.41	NS
HDL cholesterol (mmol/l)	1.37 ± 0.64	1.20 ± 0.36	NS
TG (mmol/l)	1.39 ± 0.90	1.95 ± 1.12	NS

*BMI* body mass index, *DM* diabetes mellitus, *eGFR* estimated glomerular filtration rate, *ACEI* angiotensin-converting enzyme inhibitor, *ARB* angiotensin receptor blocker, *BB* beta-blocker, *CCB* calcium channel blocker, *CAD* coronary artery disease, *LVEF* left ventricular ejection fraction, *LVMI* left ventricular mass index, *LVM* left ventricular mass, *DD* diastolic dysfunction, *E/A* mitral inflow, *EDT* mitral inflow E wave deceleration time, *Ea* early diastolic transmitral pulse-wave Doople flow, *Aa* late (atrial) transmitral pulse-wave Doppler flow, *Hb* hemoglibin, *UA* uric acid, *HDL* high-density lipoprotein, *TG* triglyceride

**Fig. 2:**
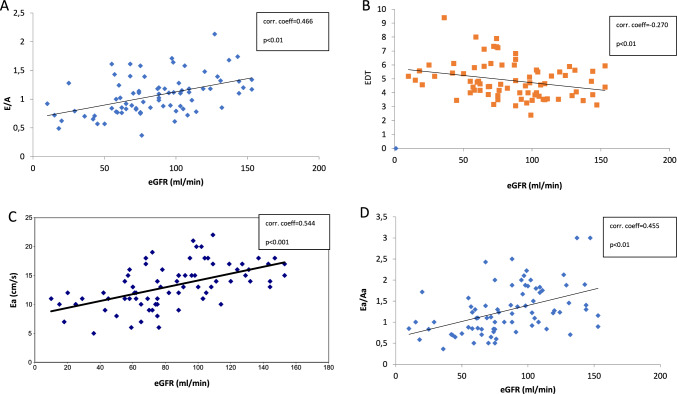
Correlations between diastolic function parameters, tissue Doppler parameters, and eGFR

There was a significant correlation between eGFR and diastolic function parameters: mitral inflow E and A wave ratio (E/A) (r = 0.466; p < 0.01), E wave deceleration time (EDT) (r = − 0.270; p < 0.01), and tissue Doppler image parameters: early diastolic mitral annular velocity (Ea) (r = 0.544; p < 0.01), late diastolic mitral annular velocity ratio (Ea/Aa) (r = 0.455; p < 0.01) (Fig. 2).

**Fig. 3:**
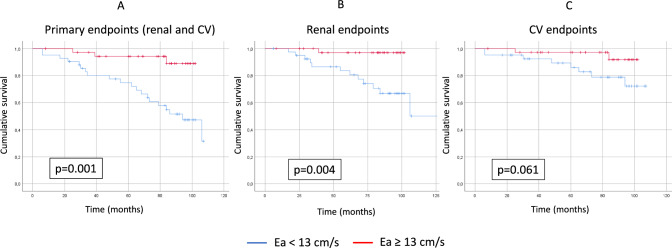
Kaplan–Meier curves based on Ea in IgAN in primary combined (**A**), secondary renal (**B**), and secondary cardiovascular endpoints (**C**)

Patients with decreased Ea (< 13 cm/s) had significantly more endpoint events (20/42 patients versus 3/37 patients, Chi-square: 11.449; p = 0.001 by the Mantel-Cox log-rank test) than patients with higher Ea (≥ 13 cm/s). Analyzing the endpoints separately (cardiovascular or renal), the renal endpoint was significant (Chi-square: 8.441; p = 0.004), but the cardiovascular endpoint was not significant (Chi-square: 3.506; p = 0.061) (Fig. 3). The independent factor that influenced Ea was only EDT by univariate and multivariate regression (Table 2), and the independent factors that influenced Ea were age and albuminuria by logistic regression (Table 3). Using the Cox regression model, the primary endpoint independent predictors of survival were gender, eGFR, diabetes, dyslipidemia, Ea, and E/Ea (Table 4). The secondary renal endpoint predictors were gender, eGFR, dyslipidemia, urine albuminuria, and Ea. The secondary CV endpoint predictors were diabetes, eGFR, E/A, E/Ea, Ea, and Aa.

**Table 2:** Uni- and multivariate regression analysis influencing factors for Ea

Parameters	Univariate analysis					Multivariate analysis						
	B	Std. errors	Beta	t	p	B	Std. errors	Beta	t	p	95% CI for B lower	95% CI for B upper
Age	− 0.179	0.032	− 0.548	− 5.678	** < 0.001**	− 0.060	0.043	− 0.182	− 1.392	0.170	− 0.147	0.027
Gender	− 0.587	0.885	− 0.076	− 0.663	0.510	0.569	0.845	0.074	0.674	0.503	− 1.125	2.264
eGFR	0.059	0.011	0.544	5.621	** < 0.001**	0.017	0.016	0.160	1.108	0.273	− 0.014	0.049
HT	− 4.231	0.862	− 0.493	− 4.909	** < 0.001**	− 0.681	0.982	− 0.079	− 0.694	0.491	− 2.649	1.287
BMI	− 0.253	0.086	− 0.324	− 2.927	**0.005**	− 0.109	0.086	− 0.138	− 1.262	0.212	− 0.282	0.064
Dyslipidemia	− 2.420	0.814	− 0.325	− 2,974	**0.004**	− 1.014	0.926	− 0.137	− 1.096	0.278	− 2.871	0.842
IgAN duration	− 0.003	0.045	− 0.008	− 0.066	0.948	− 0.008	0.043	− 0.019	− 0.176	0.861	− 0.095	0.080
Smoking	− 1.669	1.124	− 0.169	− 1.485	0.142	− 0.961	0.978	− 0.099	− 0.982	0.330	− 2.923	1.000
DM	− 3.000	0.880	− 0.366	− 3.410	**0.001**	− 0.696	0.878	− 0.084	− 0.793	0.431	− 2.457	1.065
Hb	0.083	0.277	0.035	0.300	0.765	− 0.091	0.256	− 0.038	− 0.355	0.724	− 0.603	0.422
UA	− 0.020	0.005	− 0.418	− 3.982	** < 0.001**	− 0.004	0.006	− 0.078	− 0.683	0.497	− 0.015	0.007
Chol	− 0.701	0.354	− 0.223	− 1.980	0.051	− 0.206	0.317	− 0.068	− 0.650	0.518	− 0.842	0.430
HDL	0.080	0.822	0.011	0.098	0.923	− 0.192	0.797	− 0.027	− 0.241	0.811	− 1.789	1.406
TG	− 0.755	0.401	− 0.212	− 1.882	0.064	0.284	0.463	0.081	0.614	0.542	− 0.644	1.212
Urine albumin	− 0.001	0.001	− 0.245	− 2.188	**0.032**	− 0.001	0.001	− 0.141	− 1.413	0.163	− 0.002	0.000
LVEF	0.014	0.065	0.025	0.216	0.830	0.071	0.058	0.125	1.217	0.229	− 0.046	0.187
LVMI	− 0.075	0.016	− 0.467	− 4.570	** < 0.001**	− 0.009	0.022	− 0.055	− 0.414	0.681	− 0.053	0.035
EDT	− 0.039	0.010	− 0.431	− 4.107	** < 0.001**	− 0.021	0.010	− 0.232	− 2.112	**0.039**	− 0.041	− 0.001

*eGFR* estimated glomerular filtration rate, *HT* hypertension, *DM* diabetes mellitus, *BMI* body mass index, *Hb* hemoglobin, *UA* uric acid, *Chol* cholestrol, *HDL* high density lipoptrotein cholesterol, *TG* triglyceride, *LVEF* left ventricular ejection fraction, *LVMI* left ventricular mass index, *E* wave decelaration time

p < 0.05

**Table 3:** Uni- and multivariate logistic regression analysis influencing factors for Ea

Parameter	Univariate logistic regression								Multivariate logistic regression							
	B	S.E	Wald	df	p	Exp (B)	CI 95% lower	CI 95% upper	B	S.E	Wald	df	p	Exp (B)	CI 95% lower	CI 95% upper
Age	− 0.313	0.142	4.861	1	**0.027**	0.731	0.553	0.966	− 0.146	0.036	15.995	1	** < 0.001**	0.864	0.805	0.928
Gender	− 2.513	1.783	1.985	1	0.159	0.081	0.002	2.671	0.530	0.471	1.270	1	0.260	1.700	0.676	4.275
eGFR	− 0.023	0.035	0.454	1	0.500	0.977	0.913	1.046	0.044	0.011	17.259	1	** < 0.001**	1.045	1.023	1.067
HT	4.153	2.144	3.754	1	0.053	63.647	0.953	4251.631	2.833	0.796	12.665	1	** < 0.001**	17.000	3.571	80.931
BMI	0.122	0.144	0.718	1	0.397	1.130	0.852	1.500	− 0.097	0.052	3.432	1	0.064	0.908	0.820	1.006
Dyslipidemia	3.445	1.954	3.109	1	0.078	31.353	0.681	1443.752	1.479	0.488	9.188	1	**0.002**	4.387	1.686	11.415
IgANP duration	0.078	0.065	1.461	1	0.227	1.082	0.952	1.228	0.001	0.024	0.001	1	0.994	1.000	0.954	1.048
Smoking	1.266	2.217	0.326	1	0.568	3.545	0.046	273.302	0.195	0.594	0.108	1	0.742	1.216	0.379	3.898
DM	0.668	1.366	0.239	1	0.625	1.951	0.134	28.39	1.471	0.575	6.552	1	**0.010**	4.352	1.411	13.419
Hb	− 0.443	0.482	0.842	1	0.359	0.642	0.250	1.654	0.091	0.148	0.383	1	0.536	1.096	0.820	1.463
UA	− 0.012	0.011	1.060	1	0.303	0.988	0.967	1.011	− 0.010	0.003	8.998	1	**0.003**	0.990	0.983	0.996
Chol	0.275	0.500	0.303	1	0.582	1.317	0.494	3.506	− 0.161	0.190	0.714	1	0.398	0.851	0.586	1.237
HDL	− 0.475	1.694	0.079	1	0.779	0.622	0.022	17.212	0.459	0.492	0.871	1	0.351	1.582	0.604	4.146
TG	− 0.245	0.989	0.062	1	0.804	0.783	0.113	5.432	− 0.615	0.273	5.090	1	**0.024**	0.540	0.317	0.922
Urine albumin	− 0.003	0.001	3.947	1	**0.047**	0.997	0.995	1.000	− 0.001	0.000	3.997	1	**0.046**	0.999	0.998	1.000
LVEF	0.255	0.148	2.965	1	0.085	1.290	0.965	1.724	0.019	0.035	0.308	1	0.579	1.019	0.953	1.091
LVMI	− 0.027	0.045	0.363	1	0.547	0.973	0.891	1.063	− 0.065	0.017	14.978	1	** < 0.001**	0.937	0.906	0.968
EDT	− 0.044	0.023	3.619	1	0.057	0.957	0.915	1.001	− 0.018	0.007	6.599	1	**0.010**	0.983	0.970	0.996

*eGFR* estimated glomerular filtration rate, *HT* hypertension, *DM* diabetes mellitus, *BMI* body mass index, *Hb* hemoglobin, *UA* uric acid, *Chol* cholestrol, *HDL* high density lipoptrotein cholesterol, *TG* triglyceride, *LVEF* left ventricular ejection fraction, *LVMI* left ventricular mass index, *E* wave decelaration time

p < 0.05

**Table 4:** Cox regression analysis of primary combined and secondary renal and cardiovascular endpoints influencing parameters

Primary combined endpoints								
	B	SE	Wald	df	p	Exp (B)	95% CI for lower	95% CI for upper
Gender	− 2.310	0.782	8.722	1	**0.003**	0.099	0.021	0.460
Age	0.004	0.030	0.022	1	0.881	1.004	0.948	1.064
HT	− 1.112	1.352	0.676	1	0.411	0.329	0.023	4.655
DM	− 1.556	0.633	6.039	1	**0.014**	0.211	0.061	0.730
BMI	0.040	0.065	0.383	1	0.536	1.041	0.916	1.183
eGFR	− 0.027	0.010	7.960	1	**0.005**	0.973	0.955	0.992
Dyslipidemia	− 1.718	0.761	5.100	1	**0.024**	0.179	0.040	0.797
Urine albumin	0.001	0.001	3.737	1	0.053	1.001	1.000	1.001
Ea	− 0.280	0.141	3.944	1	**0.047**	0.756	0.574	0.996
E/A	1.588	1.432	1.229	1	0.268	4.892	0.295	81.020
E/Ea	− 0.536	0.260	4.241	1	**0.039**	0.585	0.351	0.974
Aa	0.101	0.104	0.937	1	0.333	1.106	0.902	1.356
Secondary renal endpoints								
Gender	− 2.454	1.109	4.898	1	**0.027**	0.086	0.010	0.755
Age	0.001	0.040	0.001	1	1.000	1.000	0.925	1.081
HT	− 2.395	1.862	1.654	1	0.198	0.091	0.002	3.509
DM	− 1.100	0.832	1.749	1	0.186	0.333	0.065	1.699
BMI	0.064	0.099	0.424	1	0.515	1.066	0.879	1.294
eGFR	− 0.040	0.014	7.633	1	**0.006**	0.961	0.934	0.988
Dyslipidemia	− 2.651	1.303	4.136	1	**0.042**	0.071	0.005	0.908
Urine albumin	0.002	0.001	9.279	1	**0.002**	1.002	1.001	1.003
Ea	− 1.152	0.495	3.910	1	**0.045**	0.859	0.586	1.258
E/A	− 0.509	2.059	0.061	1	0.805	0.601	0.011	33.975
E/Ea	− 0.162	0.340	0.227	1	0.634	0.850	0.437	1.656
Aa	− 0.171	0.179	0.911	1	0.340	0.843	0.594	1.197
Secondary CV endpoint								
Gender	− 2.695	1.434	3.532	1	0.060	0.068	0.004	1.122
Age	− 0.026	0.053	0.239	1	0.625	0.974	0.878	1.081
HT	− 9.669	318.615	0.001	1	0.976	0.000	0.000	1.014
DM	− 3.196	1.389	5.291	1	**0.021**	0.041	0.003	0.623
BMI	0.056	0.098	0.322	1	0.570	1.057	0.872	1.282
eGFR	− 0.053	0.022	5.833	1	**0.016**	0.948	0.908	0.990
Dyslipidemia	− 2.008	1.199	2.807	1	0.094	0.134	0.013	1.406
Urine albumin	− 0.002	0.001	2.272	1	0.132	0.998	0.996	1.001
Ea	− 0.655	0.239	7.543	1	**0.006**	0.519	0.325	0.829
E/A	4.876	2.004	5.918	1	**0.015**	131.040	2.579	6657.401
E/Ea	− 2.070	0.862	5.767	1	**0.016**	0.126	0.023	0.683
Aa	0.573	0.261	4.832	1	**0.028**	1.774	1.064	2.956

*HT* hypertension, *DM* diabetes mellitus, *BMI* body mass index, *eGFR* estimated glomerular filtration rate, *Ea* early diastolic transmitral pulse-wave Doople flow, *E/A* mitral inflow, *Aa* late (atrial) transmitral pulse-wave Doppler flow, *E/Ea* early mitral inflow/early diastolic transmitral pulse-wave Doppler flow

p < 0.05

## Discussion

Our study examined the relationship of Doppler and tissue Doppler echocardiography parameters with renal function in a homogenous immunocomplex-mediated CKD population of IgAN patients, and we found a correlation between E/A, EDT Ea, Ea/A, and eGFR.

Based on our results, in IgAN, the increase in diastolic dysfunction is observed with decreasing renal function, which is best described by EDT and Ea as defined by TDI, similarly in CKD.

Cardiovascular alterations develop in early CKD stages (echocardiographic parameters). There may be a difference in the dynamics of this, which may stem from the etiology of the disease [18], but there is a small amount of data in the literature on this. Patients at high CV risk should be screened in the early stages of CKD. TDI parameters are sensitive early markers, supported by a close correlation with eGFR, even in relatively few cases.

Previous studies have shown that E/Ea, an estimate of LV filling pressure by Doppler echocardiography, is a predictor of all-cause mortality in patients with LV systolic dysfunction and after acute myocardial infarction [19, 20]. Another study in patients with ESRD also reported that an E/Ea ≥ 15 could predict an increase in LV filling pressure with a sensitivity of 82% and a specificity of 88% and was associated with an increased risk of mortality [21]. In addition to predicting all-cause mortality, a high E/Ea has been reported to provide additional prognostic value in patients with ESRD beyond traditional echocardiographic parameters [21, 22]. Chen et al. also found that a high E/Ea was associated with an increased risk of CV events in patients with CKD [23].

A previous study evaluated the association between E/Ea and the progression of renal dysfunction in patients with and without CKD and found a higher E/Ea in the patients with a more rapid decline in renal function [24]. Chen et al. also reported an independent association between a high E/Ea and an increased risk of starting dialysis in patients with CKD stages 3–4 [25]. This implies that a high E/Ea ratio may lead to high volume status, increase renal efferent pressure, and decrease renal blood flow, subsequently leading to a progressive decline in renal function [25, 26]. A higher preload status may also contribute to a more rapid progression to dialysis.

In the traditional pathophysiological model, pressure overload leads to concentric LV hypertrophy, fibrotic remodeling, and LVDD. Eventually, LVDD leads to left atrial (LA) hypertension and remodeling, pulmonary venous hypertension, and right ventricular and atrial remodeling.

In the other model, proinflammatory cardiovascular and noncardiovascular coexisting conditions lead to systemic microvascular endothelial inflammation, global cardiac and skeletal muscle inflammation, and subsequent fibrosis. Thus, systemic microvascular endothelial inflammation has been proposed as an additional mechanism leading to myocardial inflammation and fibrosis, increasing oxidative stress, and promoting alterations in cardiomyocyte signaling pathways. These changes promote cardiomyocyte remodeling as well as microvascular dysfunction in cardiac and skeletal muscle [27]. The main pathophysiological alteration leading to HFpEF remains incompletely defined.

In CKD, several conditions contribute to the pathogenesis of HFpEF, such as arterial hypertension [28]. One of the most significant cardiac alterations in CKD is left ventricular hypertrophy (LVH), and CKD contributes significantly to its development. It develops early in the progression of kidney dysfunction, is frequently accompanied by myocardial fibrosis and LVDD, and is an independent risk factor for mortality in this population. The role of CKD is well documented, but its basis is not fully understood. The effect of uremia on the myocardium includes structural changes such as cardiomyocyte hypertrophy, myocardial fibrosis, and thickening of the intramural arteries. Together, these structural changes predispose to LVDD in response to the cumulative action of traditional and CKD-related risk factors [29–31]. There is good evidence that interstitial fibrosis is related to changes in collagen myocardial metabolism. On the other hand, cardiomyocyte hypertrophy and vascular remodeling may be adaptive responses to pressure and volume overload [29]. Others, such as hyperphosphatemia, hyperparathyroidism, and hypovitaminosis D, play a greater role in more advanced stages of CKD and dialysis [32, 33]. Another important factor is renin–angiotensin–aldosterone system (RAAS) activation, potentially inducing myocardial fibrosis and hypertrophy. Activation of the intracardiac RAAS seems to be critically involved in the overload status observed in dialysis, but angiotensin II and aldosterone can also be involved in myocardial cell hypertrophy and fibrosis independent of afterload [34].

In Escoli et al.’s review, they suggest patients with CKD and ESRD should be monitored regularly (perhaps every 1–2 years) for the development and assessment of the severity of LVH and cardiac fibrosis, most likely with serial echocardiography [35]. Nevertheless, our data suggest that it may also be useful for CKD 1–3 stages.

It is well known that hypertension is a major risk factor for the development of LVDD in chronic hemodialyzed patients [36]. However, in the early stages of CKD, when the blood pressure elevation is not so significant, the mechanism of the relationship between CKD and LVDD is not fully characterized. Therefore, the value of the echocardiography examination should be important in CKD.

Nerpin et al. [37] identified a significant inverse relationship between eGFR and LVDD in a community-based elderly population. LVDD is common in CKD patients, even in the early stages [38]. Several echocardiographic parameters, including LVH, large left atrial volume, and decreased LV ejection fraction (LVEF), have been revealed to be associated with cardiac events among patients with CKD [39, 40]. Furthermore, CKD at this age was reported to be strongly correlated with adverse cardiovascular (CV) outcomes [40]. However, LVDD was not associated with incident heart failure (HF) or all-cause death in the CRIC study [41]. These discrepancies have arisen because the studies used different parameters to evaluate cardiac function, particularly LV diastolic dysfunction.

In Shu’s KNOW-CKD study, LVDD was independently associated with adverse CV outcomes and all-cause mortality in patients with predialysis CKD [42].

Liang et al. proved that systolic dysfunction and LVDD demonstrated mutually augmentative effects on CV mortality and suggested that, together with conventional nephroprotection, early cardioprotection should be emphasized for patients with CKD in the early stage. Therefore, cardioprotective management should be initiated as early as possible after CKD diagnosis [43].

Known CV risk factors such as baseline eGFR, proteinuria, and hypertension are also risk factors for CKD progression, contributing to the acceleration of renal function loss and progression to ESKD. However, the progression of CKD, which is a complex process, cannot be explained in all cases by these traditional risk factors.

In our study, we analyzed the prognostic role of LVDD for CV and renal endpoints. Based on the correlation between Ea and GFR in IgAN, decreased Ea and the development of LVDD may predict the progression of renal disease and CV events before reaching ESRD. In our IgAN patients, we found that the decreased Ea had a significant effect on both combined and individual renal and CV outcomes.

We hypothesize that a decreased Ea due to LVDD development and a decreased eGFR may synergistically affect the poor prognosis. Worsening renal function, a higher incidence of LVDD, and CV complications result in a worse prognosis and impaired LVDD (lower Ea). As a result of all this, a greater deterioration in renal function is expected. However, the clear connection between the deterioration of kidney function and LVDD is not elucidated.

LVDD is closely linked to CKD because the uremic milieu predisposes patients with CKD to systemic arterial stiffness and myocardial interstitial fibrosis, ultimately leading to LVH and impaired LV relaxation and compliance [44, 45].

More than half of the patients with stages 1 and 2 CKD had a normal diastolic function, whereas only 13% of those with stage 5 CKD had a normal diastolic function. This relationship between CKD progression and LVDD burden is consistent with that observed in a population study [46].

The association of LVDD with all-cause mortality remains controversial in CKD. The CRIC investigators revealed that LVDD was not associated with incident HF or all-cause mortality [42]. However, the CRIC study did not use tissue Doppler in the assessment of LVDD. Therefore, it is not surprising that no association was found between LVDD and outcomes in the CRIC analysis. In contrast, the Mayo Clinic group demonstrated a significant association between LVDD and all-cause mortality in patients with CKD; nonetheless, no interaction between LVDD and CKD stage has been identified [46, 47].

Hypertension is a very common complication in IgAN [47, 48], affecting 50–70% of patients, and this was confirmed by our data. Increased RAAS activity and hypertension in CKD also increase the incidence of vascular events; thus, RAAS blockade is the standard treatment (recommended in all guidelines) in these patients in general and for the patients who have IgAN [49–51]. Based on our former results and those of others, we thought that RAAS also plays a key role in the development of arterial stiffness and LVDD in renal disease, as in IgAN [49–51]. However, there is no data on whether ACEI and/or ARB treatment could afford an LVDD-lowering effect in patients with IgAN. In our study, at the start, more patients received ACEI and/or ARB therapy in the lower Ea group than in the higher Ea group. However, we were unable to distinguish between RAAS inhibitor users and non-users. At the end of the observation, almost all patients received RAAS blockers.

In our study, there was a significant difference in the use of a RAAS inhibitor between lower and higher Ea patients. Based on this observation, RAAS may be important in the evaluation of LVDD. However, it should be noted that the blood pressure of the study population was well controlled. Patients with IgAN exhibiting lower Ea had deteriorated renal function and an increased incidence of ESKD and CV complications in both sexes compared to those with higher Ea values. However, this may be particularly important because the further progression of CKD may be accelerated in older age and with impaired renal function, and more complications may develop with worse CV status, which may also worsen the prognosis by launching a "vicious circle".

Metabolic parameters play an important role in the progression of IgAN, as has been previously demonstrated by our workgroup and also by others [52, 53]. According to our findings, the appearance of metabolic syndrome in CKD is known to worsen the prognosis, and if the TDI parameters worsen, LVDD develops; these alterations cause a worsening in the metabolic parameters (and vice versa). It follows from all this that these parameters should be treated as soon as possible, which confirms the importance of complex metabolic risk reduction in these patients [52, 53].

### Limitations of the study

Our results indicated that the Ea value obtained from echocardiography has prognostic significance; however, difficulties may occur during echocardiographic measurement. In some populations, specifically the elderly, a lack of cooperation can be a problem. Renal function was determined by estimating GFR, which is widely accepted in the literature, but the limitations of the formula are also well known. The extent and change of proteinuria were not examined in the present study. The evaluation of the results may also be weakened by the low number of cases, especially the low number of female patients. Our study follow-up time was not long enough to get significant results for the CV endpoints. We did not examine left or right atrial volume, myocardial strain, or strain rate, and we did not use this parameter for LVDD determination. In our study, there was no control group to compare our results with a non-CKD population. Despite these limitations, the results of this study highlight that the onset of target organ damage in CKD is predicted by decreased Ea.

## Conclusion

Our results suggest that Ea as assessed by tissue Doppler echocardiography appears suitable for estimating prognosis in IgAN patients and that the lower value is an independent prognostic factor for ESKD and CV events, but there was no control group without IgA nephropathy, so it cannot be concluded that this was specific for this entity. Lower Ea should call attention to those CKD patients who have higher renal and CV risk at an earlier stage of CKD (G2-3) and need to be monitored more closely, referred for further CV tests, and given maximal nephroprotection.

Our findings support the role of echocardiography in the high-CV-risk population of CKD patients, which also helps to understand the relationship between heart abnormalities and renal impairment.

In conclusion, impaired renal function gradually correlates with LVDD and tissue Doppler parameters in patients with IgAN. Decreased renal function is associated with decreased Ea and LVDD, which are responsible for poor prognosis due to worse CV and renal outcomes. In the background, the role of common vascular and myocardial pathological remodeling, which is exacerbated by metabolic changes, could be hypothesized. To confirm our results, further large-scale, multicenter prospective studies are warranted to evaluate the role of CV risk factors in mediating the changes in the TDI parameter, Ea, as well as the complex relationships between CV disease and CKD.

## Data Availability

The datasets used and analyzed during the current study are available from the corresponding author upon reasonable request.
